# Comparison of ruminal microbiota, *IL-1β* gene variation, and tick incidence between Holstein × Gyr and Holstein heifers in grazing system

**DOI:** 10.3389/fmicb.2024.1132151

**Published:** 2024-02-26

**Authors:** Daiana Francisca Quirino, Marcos Inácio Marcondes, Kellen Ribeiro de Oliveira, Simone Elisa Facioni Guimarães, Juliana Soares da Silva, Garret Suen, Letícia Elisa Rossi, Camila Soares Cunha, Hilario Cuquetto Mantovani, Polyana Pizzi Rotta

**Affiliations:** ^1^Department of Animal Science, Universidade Federal de Viçosa, Viçosa, Minas Gerais, Brazil; ^2^Department of Animal Science, Washington State University, Pullman, WA, United States; ^3^Department of Microbiology, Universidade Federal de Viçosa, Viçosa, Minas Gerais, Brazil; ^4^Department of Bacteriology, University of Wisconsin, Madison, WI, United States; ^5^School of Veterinary Medicine and Animal Science, Universidade Federal de Mato Grosso do Sul, Campo Grande, Mato Grosso do Sul, Brazil; ^6^Department of Animal and Dairy Sciences, University of Wisconsin, Madison, WI, United States

**Keywords:** crossbred heifer, Guinea grass, heat stress, pasture, rumen microbiology

## Abstract

**Introduction:**

The variation in bacterial communities among breeds has been previously reported and may be one of the reasons why Holstein × Gyr dairy heifers have better development in grazing systems in tropical conditions. This study aimed to explore the ruminal microbiota composition, the *IL-1β* gene variation, tick incidence, and blood parameters of Holstein × Gyr (½ Holstein × ½ Gyr) and Holstein heifers grazing intensely managed Guinea grass (*Panicum maximum* Jacq. cv. Mombaça).

**Methods:**

Sixteen heifers were divided into two groups consisting of 8 Holstein × Gyr and 8 Holstein heifers. The experimental period was comprised of 3 periods of 21 days. Ruminal samples were taken via the stomach tube technique. The sequencing of the V4 hypervariable region of the 16S rRNA gene was performed using the Illumina MiSeq platform. Counting and collection of ticks were conducted each 21 days. Blood and skeletal muscle tissue biopsies were performed at the end of the experiment.

**Results:**

Firmicutes were the most abundant phyla present in both breed rumen samples and Bacteroidota showed differences in relative abundance between breed groups, with greater values for Holstein heifers (*p* < 0.05 with FDR correction). The 10 most abundant unique OTUs identified in each breed included several OTUs of the genus *Prevotella*. Holstein heifers had a greater tick count and weight (9.8 ticks/animal and 1.6 g/animal, respectively) than Holstein × Gyr (2.56 ticks/animal and 0.4 g/animal, respectively). We found nucleotide substitutions in the *IL-1β* gene that might be related to adaptation and resistance phenotypes to tick infestation in Holstein × Gyr heifers. Blood concentrations of urea, albumin, insulin-like growth factor 1, triiodothyronine, and thyroxine were greater in Holstein × Gyr than in Holstein heifers.

**Conclusion:**

Adaptations in Holstein × Gyr heifers such as ruminal microbiota, tick resistance, nucleotide substitutions in *IL-1β* gene, and hormone concentration suggest a better energy metabolism and thermoregulation resulting in better performance in tropical grazing systems.

## 1 Introduction

The Holstein × Gyr (½ Holstein × ½ Gyr), also known as the Girolando breed, is a popular crossbred among dairy producers in tropical regions (Mellado et al., 2011; Washburn and Mullen, 2014). The Girolando cattle allies milk production traits from Holstein and adaptability to tropical climate presented by Zebu breeds (Santos et al., 2011; Fraga et al., 2016). However, Holstein and crossbred genetics have been modified over the years (Washburn and Mullen, 2014), and the mechanisms behind different performance responses of purebred or crossbred young animals in tropical pasture-based systems are not fully understood. Abreu et al. (2022) suggested that Holstein heifers have less heat tolerance, demonstrated by the greater bite rate (20 vs. 17.3), and shorter grazing time spent in each meal compared to crossbred heifers. Therefore, the variation in performance of Holstein × Gyr and Holstein heifers might be explained by the ability to graze in high temperatures, which consequently impacts time spent in grazing and resting (Quirino et al., 2022), resistance to ectoparasites, and susceptibility to infectious diseases (Otto et al., 2018). Beyond the suggested differences above, we speculate that susceptibility to diseases and grazing behavior would also cause a significant change in the ruminal microbiota, which could impact the intake and performance of grazing animals (Uyeno et al., 2010).

The variation in bacterial communities among breeds has been previously reported (Uyeno et al., 2010; Paz et al., 2016). In addition, heat stress may impact the population of microorganisms essential for fiber digestion, and these changes are responsible for regulating nutrient cycling in the host (Church, 1993), consequently impacting animal performance and metabolism.

In addition to heat stress responses, Holstein animals are more susceptible to tick infestation because the physical barriers, including skin thickness, the density of fur coat, and odor, as well as the self-cleaning ability with larger space between tongue papillae are less effective in removing ticks in the larvae stage (Tabor et al., 2017). Infestations by ectoparasites such as the bovine tick *Rhipicephalus* (*Boophilus*) *microplus* may impact animal/herd performance due to secondary illnesses (Léger et al., 2013), and can even lead to animal death. Over 1 g in beef cattle weight might be lost daily for each engorging tick (Jonsson, 2006). The *IL-1β* gene is associated with the immune response and has been shown to be increased during tick infestation (Piper et al., 2008; Brannan et al., 2014; Thutwa et al., 2021). Greater polymorphism in the *IL-1β* gene may impact the animals’ adaptive response to ticks, compromising the cascade of pro-inflammatory cytokines (Torina et al., 2020), activation of endothelium, and local upregulation of cell-surface adhesion (Rahman et al., 2016). Tick infestation is associated with lower productivity and decreased efficiency of feed utilization (Berman, 2011), and the Holstein animals are considered more susceptible to ticks (Glass and Coussens, 2005) in tropical grazing systems.

In addition, diet composition can induce significant changes in rumen microbiota, which in turn, is also influenced by host genetics (Liu et al., 2021). Menezes et al. (2011) observed a predominance of Prevotellaceae, Erysipelotrichaceae, and Veillonellaceae in grazing cows, while cows fed a total mixed ration had a predominance of Fibrobacteriaceae. However, a comparison between ruminal microbiota from crossbred (Holstein × Gyr) and purebred Holstein heifers in grazing systems has not yet been demonstrated, which might help explain the differences in digestibility, beyond feeding behavior, reported in previous studies (Oliveira Filho et al., 2018; Silvestre et al., 2021; Quirino et al., 2022). Thus, changes in ruminal microbiota and tick infestation potentially reduce the productivity of grazing animals, especially in Holstein animals. Therefore, for tropical systems, crossing taurine with zebu breeds can increase rusticity while maintaining production (Berman, 2011).

We hypothesized that Holstein × Gyr heifers perform better than Holstein heifers in grazing systems due to differences in the rumen microbiota, polymorphisms in the *IL-1β* gene, lower tick incidence, and greater concentrations of specific blood hormones that indicate better adaptability to tropical conditions. Therefore, this study aimed to evaluate the ruminal microbiota composition, polymorphisms in the *IL-1β* gene, tick incidence, and blood parameters from Holstein × Gyr and Holstein heifers managed in an intermittent grazing system of Guinea grass (*Panicum maximum* Jacq. cv. Mombaça) in tropical conditions.

## 2 Materials and methods

### 2.1 Experimental design and animal management

The study was conducted at the Universidade Federal de Viçosa (Viçosa, MG, Brazil), located in the Southwest region of Minas Gerais State (20,7604400S, 42,8,608,200 W), Brazil. The experiment was conducted during the summer season, from December 2016 to April 2017. The experiment was conducted following the standard procedures for Animal Care and Handling stated in the Universidade Federal de Viçosa guidelines process number 24/2018. Data on weather conditions were obtained from the Local Weather Station located 1.39 km from the experimental area. The experiment was composed of three periods of 21 days after an adaptation period to the diet and management of 45 days in a grazing system of Guinea grass. Before the trial, all animals received a preventive application of an anti-parasitic (Eprinex Pour-On, Boehringer Ingelhein, São Paulo, São Paulo, Brazil) at 1 mL/10 kg body weight (BW). Sixteen heifers were used in this trial, and they were divided into four groups (*n* = 4) composed of four Holstein and four Holstein × Gyr (½ Holstein × ½ Gyr) heifers with two different BW assigned in a randomized block design with a repeated measure scheme. The groups averaged 258.6 ± 24.80 kg and 157.1 ± 24.99 kg BW and grazed two separate sets of 16 paddocks each. Blocks were considered a combination of a set of paddocks and animals’ weights and treatments were randomized within each block (set of paddocks 1 had the heavier animals and set of paddocks 2 had the lighter animals).

Animals were managed in a rotational grazing system comprising 32 Guinea grass (*Panicum maximum* Jacq. cv. Mombaça) paddocks with an 800 m^2^ area, fertilized with 200 kg of N/ha/yr. and 150 kg of K_2_O/ha/yr., for 1 d grazing period. Each set of paddocks had a feeding area with a feed bunk, drinking fountains, and 4 m^2^/animal of shade freely accessed by the animals. Each heifer group received a concentrate supplement (0.5% BW on DM basis) at 1200 h and mineral salt *ad libitum*. The concentrate composition is presented in Supplementary Table S1, and was manufactured in an animal feed factory at the Universidade Federal de Viçosa.

### 2.2 Ruminal sampling, DNA extraction, and sequencing

On the last day of each period, ruminal samples were taken without fasting at 0800 h using the stomach tube technique (Lodge-Ivey et al., 2009; Henderson et al., 2013; Kumar et al., 2015). The initial volume of rumen fluid (~200 mL) was discarded to avoid saliva contamination. A sample of approximately 50 mL was stored at −80°C for DNA extraction.

Genomic DNA was extracted from ruminal samples as described by Stevenson and Weimer (2009), using the phenol/chloroform method with bead beating for mechanical disruption of microbial cells. Samples were processed and sequenced separately for each experimental period. The V4 hypervariable region of the 16S rRNA gene was paired-end sequenced on the Illumina MiSeq platform following the manufacturer’s guidelines (Illumina, Inc., San Diego, California, United States) at the University of Wisconsin-Madison (United States).

After sequencing, raw reads were demultiplexed and adapters were removed in Illumina Miseq software. Afterward, the sequences were filtered, and chimeras were removed, as well as reads shorter than 200 bp or longer than 500 bp. In addition, ambiguous sequences and homopolymers with more than 8 bp were removed. The paired-end sequences were joined, filtered, and cleaned using Mothur v.1.47.0 following MiSeq SOP (Schloss et al., 2009). V4 sequences were aligned using the SILVA v.138 16S rRNA gene reference database (Quast et al., 2012). Lastly, sequences were grouped into operational taxonomic units (OTUs) using uncorrected pairwise distances clustered with the furthest neighbor method based on a similarity cut-off of 97%. OTUs were normalized across samples, using the sample with the lowest number of sequences. For the bioinformatic analyses, sequences of the same treatment collected from three experimental periods were analyzed together and results are representative of the three sampling periods. The sequences obtained for all samples in the present study were submitted to Sequence Read Archive on the National Center for Biotechnology Information^1^ under the accession number PRJNA 956552.

### 2.3 Tissue collection, DNA extraction, and sequencing

One muscle tissue sample was biopsied at d 80 by sampling 1 g of *Longissimus dorsi* from each animal using local subcutaneous lidocaine (10 mL at 5%). Samples were washed with a sterile saline solution (0.9%) and stored overnight (4°C) in RNA Later (Qiagen, Hilden, North Rhine, Westphalia, Germany) and subsequently at −80°C. DNA was extracted using the method described by Sambrook and Russell (2001).

Primers for 2 exons of gene *IL-1β* (GenBank AY851162.1; Supplementary Table S2) were designed using Primer-BLAST. PCR was performed with GoTaq® Master Mix (Promega Corporation, Madison, WI, United States) in a Veriti™ thermocycler (Applied Biosystems, Thermo Fisher Scientific, California, United States). The temperature cycles were as follows: initial denaturation at 95°C for 2 min, 40 denaturation cycles at 95°C for 1 min, annealing at 62°C and 63°C for 1 min for primers one and two, respectively, and extension at 73°C for 1.3 min followed by a final extension at 73°C for 5 min. PCR products were purified from agarose gel using the Wizard SV Gel and PCR clean-up System kit (Promega). Amplification products were sequenced using an AB3500 Genetic Analyzer (Applied Biosystems/Thermo Fisher Scientific, California, United States). Amplicons were forward and reverse sequenced in-house on a Sanger 3,500 Genetic Analyzer platform following the manufacturer’s guidelines (Applied Biosystem Thermo Fisher Scientific, San Diego, California, United States).

Amplicon gene sequencing of the *IL-1β* gene data was processed using the BioEdit Sequence Alignment Editor version 7.0.5.3, and Muscle software version 3.8.31^2^ was used to compare the sequences obtained with the reference gene *IL-1β* sequence (GenBank AY851162.1).

### 2.4 Weighing and tick counts and collection

Heifers were weighed on three consecutive days at the beginning and the end of the experiment. Before weighing, heifers were fasted for 12 h with free access to water. After weighing, animals were placed in paddocks until the next fasting period. At the end of each experimental period, tick counts were performed on the left side of the animal’s body according to the methodology described by Wharton and Utech (1970). After tick collection, all animals received preventive treatment with a pour-on anti-parasitic (Eprinex® Pour-On, Boehringer Ingelhein, São Paulo, São Paulo, Brazil) at a dose of 1 mL/10 kg BW.

### 2.5 Blood parameters

Blood samples were collected at d 80, by jugular vein puncture using vacuum tubes (BD Vacutainer® SST® II Advance®, São Paulo, Brazil). Blood serum was separated by centrifugation (2,700 × g for 20 min at 4°C) and frozen at −20°C. Urea (K056), glucose (K082), total protein (K031), albumin (K040), and triglycerides (K117) were quantified using an automatic biochemical analyzer (Mindray, BS200E, Shenzhen, China) and Bioclin kits. IGF-1 was analyzed by chemiluminescence using the UniCell Dxl Access Immunoassay System (Beckman Coulter Inc., Brea, United States). Total T3 and total T4 were analyzed using Beckman kits (ref. number 33,830 and 33,800, respectively, Beckman Coulter®, Brea, United States) in the Access 2 Immunoassay System (Beckman Coulter Inc., Brea, United States).

### 2.6 Statistical analysis

Statistical analysis was performed using the GLIMMIX procedure of SAS (SAS University Edition) using a randomized block design model with repeated measures:


$$Y_{ijklm}=\mu +T_{i}+P_{k}+{\left(T\times P\right)}_{ik}+B_{l}+\varepsilon_{ijklm}$$

where: μ = general mean, *T_i_* = fixed effect of treatment (breed) i, *P*_k_ = fixed effect of period k, T × P_ik_ = fixed effect of the interaction between treatment *i* and period *k, B_l_ =* random effect of block *l* (combined effect of weight and paddock), and ɛ_ijklm_ = random errors with a mean 0 and variance $\sigma^{2}$ , which is the variance between measurements within animals. Fifteen covariance structures were tested for each response variable and the one that provided the lower AIC was used. Data on tick counts and weight exhibited a non-normal distribution; all available distributions in the GLIMMIX procedure were tested using the same model described above and SHIFTED T (3) distribution was used (approached normality of residues).

Data with single observations per animal were analyzed using a randomized block design model:


$$Y_{ijk}=\mu +T_{i}+B_{l}+\varepsilon_{ijk}$$

where: μ = general mean, *T_i_* = fixed effect of treatment (breed) i, *B_l_ =* random effect of block *l* (combined effect of weight and paddock), and ɛ_ijk_ = random error with a mean 0 and variance $\sigma^{2}$ .

For rumen microbiota statistical analysis, Past v.4.02 (Hammer et al., 2001) software was used to create Non-Metric Multidimensional Scaling (NMDS) plots based on the Bray–Curtis dissimilarity metric. Analysis of similarities (ANOSIM) was performed (number of permutations of 10.000) to evaluate significant differences between experimental groups using the same software. Beta diversity was also evaluated using two additional distance matrices (weighted and unweighted UniFrac) and Principal Coordinate Analysis (PCoA) as the ordination method. Permanova F-statistic and *p*-values were calculated according to Anderson (2001). Alpha diversity data were analyzed using SPSS v.22 (Norusis, 1993) for normality data using the Kolmogorov–Smirnov test. Subsequently, the Mann–Whitney non-parametric test was performed for independent samples to correlate Holstein × Gyr and Holstein data. Relative abundances of OTUs were analyzed using the Benjamini-Hochberg method to correct the false discovery rate at phylum, family, and genus levels. A two-sided White’s non-parametric t-test was performed using STAMP v.1.2.3 (Parks et al., 2014).

To determine the OTUs most likely to explain the differences between breeds, LEfSe (Linear discriminant analysis Effect Size; Segata et al., 2011) was used to identify potential biomarkers associated with the phenotypes of interest. The statistical analyses were performed in the implementation of LEfSe on the server Galaxy (version 0.1). For this purpose, a standard table of the composition of bacterial OTUs for each breed was provided.

For all analyses, significant differences were declared when *p* < 0.05, and trends when 0.05 < *p* < 0.10.

## 3 Results

### 3.1 Environmental conditions

Weather conditions varied along experimental periods, with a minimum temperature of 17.8°C and a maximum of 31.4°C. Relative humidity and Rainfall varied from 75.4% to 79.5% and 2.9 to 4.9 mm, respectively. The temperature-humidity index (THI) ranged from 68.8 to 72. Detailed information on weather data in each period was described by Quirino et al. (2022).

### 3.2 Forage allowance and chemical composition

The pre-grazing and post-grazing heights varied during the experiment, with average heights of 71.0 ± 2.91 and 43.8 ± 1.15 cm, respectively. The accumulated herbage per paddock (88.5 ± 5.13) and herbage allowance (11.0 ± 0.63) increased during periods and the major herbage accumulation occurred in periods 3 and 4 with DM of 27.4% and 25.8%, respectively, while grazing efficiency was superior (63.3%) when neutral detergent fiber was inferior (66.0%). Thus, the intake was not limited by forage allowance (Quirino et al., 2022).

### 3.3 Rumen microbiota composition

**Figure 1:**
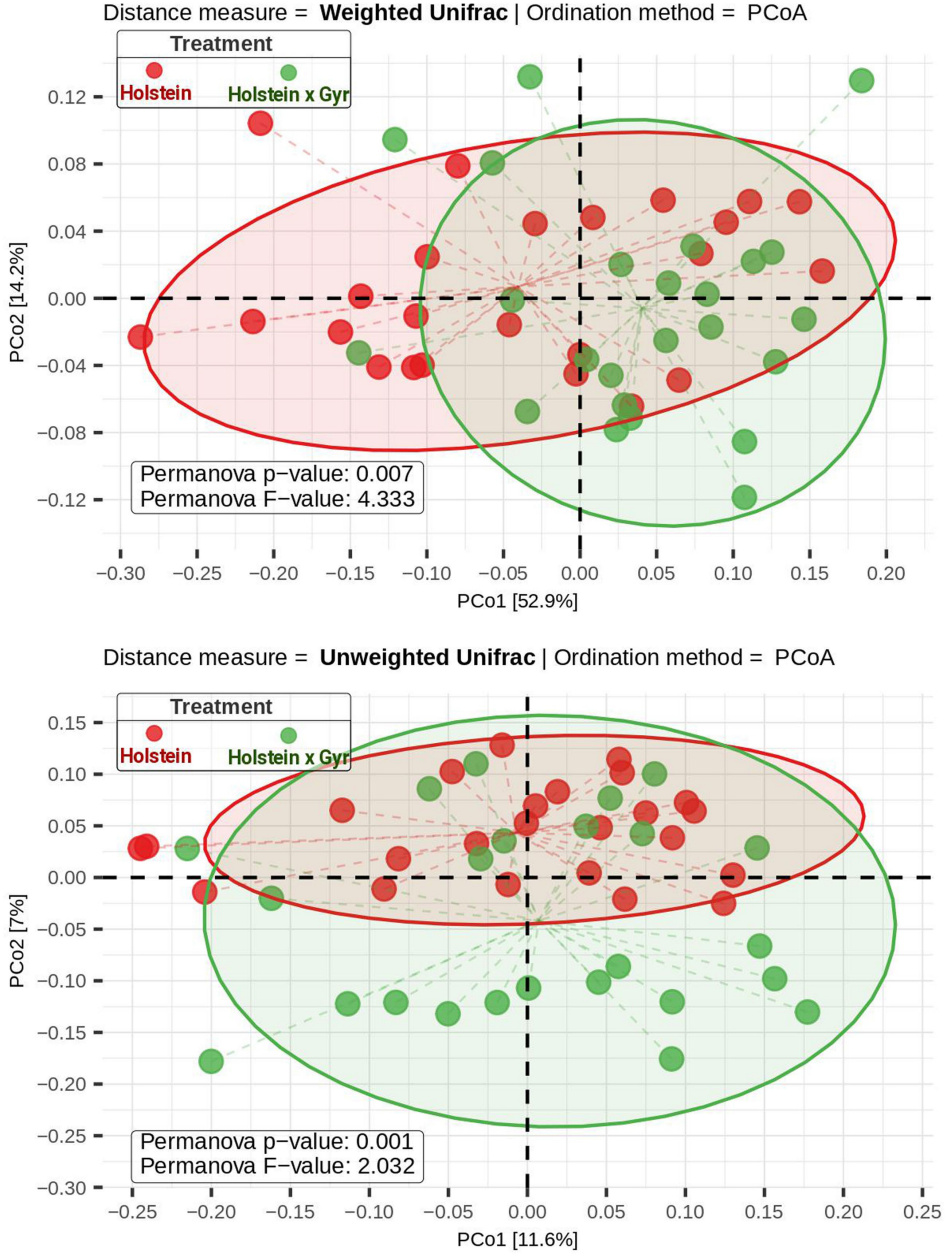
Beta diversity was evaluated using two distance matrices (weighted and unweighted UniFrac) and Principal Coordinate Analysis—PcoA as the ordination method. Permanova F-statistic and *p*-values were calculated and are shown in the PCoA plot. Holstein cattle is represented by red dots and Holstein × Gyr as green dots. Please note that some samples overlap in the plot.

After cleaning and filtering the sequencing data, 2,062.650 high-quality sequence reads were obtained in total, with a minimum Good’s coverage of 97% and a maximum value of 99% (Table 1). NMDS visualization did not show a clear separation between the microbial communities of Holstein × Gyr and Holstein heifers (Supplementary Figure 1). However, ANOSIM revealed a significant difference (*p* < 0.01) between the rumen microbiota composition of each breed (Supplementary Figure 1A). The NMDS analysis carried out separately between sampling days showed similar behavior, except for the first sampling, where no significant difference was observed between the breeds (*p* = 0.11; Supplementary Figure 1B). A significant difference between breeds was also observed (Permanova, *p* < 0.05) using weighted and unweighted UniFrac as phylogenetic distance metrics represented in a Principal Coordinate Analysis (PCoA; Figure 1). Alpha diversity metrics (Chao 1, Shannon, and Simpson indexes) were not different between breeds (*p* = 0.77, *p =* 0.48, *p* = 0.32, respectively; Supplementary Figure 2).

**Table 1:** Summary of sequencing data from ruminal samples from Holstein × Gyr and Holstein heifers (mean ± SD).

Groups		After filtering and clean-up		After normalization	
	Good’s coverage	Reads	OTUs	Reads	OTUs
Holstein	0.98 ± 0.004	44,511 ± 12,765	2,398 ± 405	20,330 ± 256	1,958 ± 273
Holstein × Gyr	0.98 ± 0.005	41,299 ± 12,617	2,344 ± 382	20,371 ± 246	1,992 ± 217

**Figure 2:**
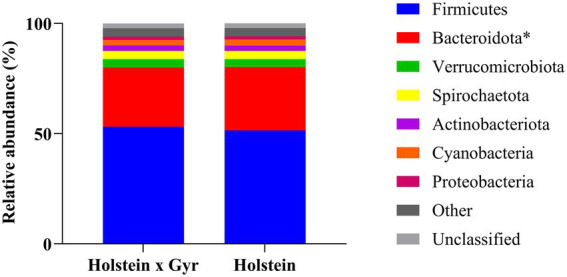
Bacterial community composition at the phylum level (relative abundances > 1.0%) in ruminal samples of Holstein × Gyr and Holstein heifers. ^*^Different with *p* < 0.05 with FDR correction using two-sided White’s non-parametric *t*-test.

The taxonomic assignment of the bacterial sequences revealed a total of 23 phyla, 41 classes, 94 orders, 156 families, and 279 genera. The phylum Firmicutes was in higher abundance for both groups, with a greater relative abundance in Holstein × Gyr heifers (53.1% ± 2.08%) compared to Holstein heifers (51.5% ± 2.13%; Figure 2). The second most abundant phylum was Bacteroidota, which was different between breeds (*p* = 0.03 with FDR correction). Relative abundances were 26.9% ± 1.92% and 28.7% ± 2.23% for Holstein × Gyr and Holstein heifers, respectively. Unclassified phyla corresponded to 2.24% ± 0.43% for Holstein × Gyr heifers and 2.10% ± 0.52% for Holstein heifers.

At the family level, Prevotellaceae and Lachnospiraceae were the predominant groups in samples from both breeds, but no significant differences were observed (*p* = 0.25 with FDR correction; Supplementary Figure 3). As for the genus level, there were no significant differences in ruminal samples from both breeds. Genus *Prevotella* showed the greatest relative abundance in both heifer groups, being 10.6 ± 1.46 for Holstein × Gyr and 11.9 ± 1.95 for Holstein (*p* = 0.33 with FDR correction; Supplementary Figure 4).

**Figure 3:**
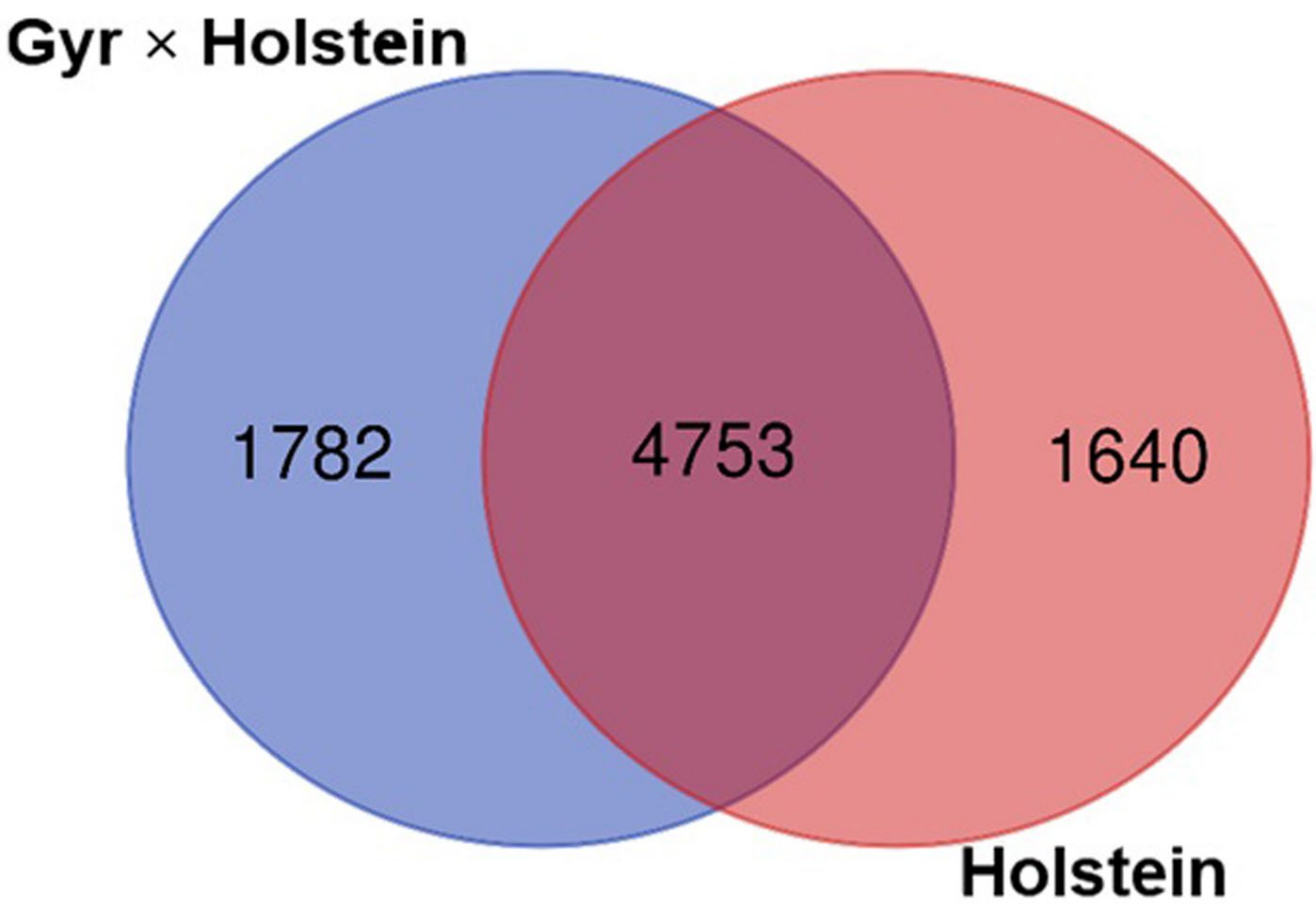
Venn diagram representing the number of OTUs that were shared between the Holstein × Gyr and Holstein breeds and those that appeared exclusively in each group.

**Figure 4:**
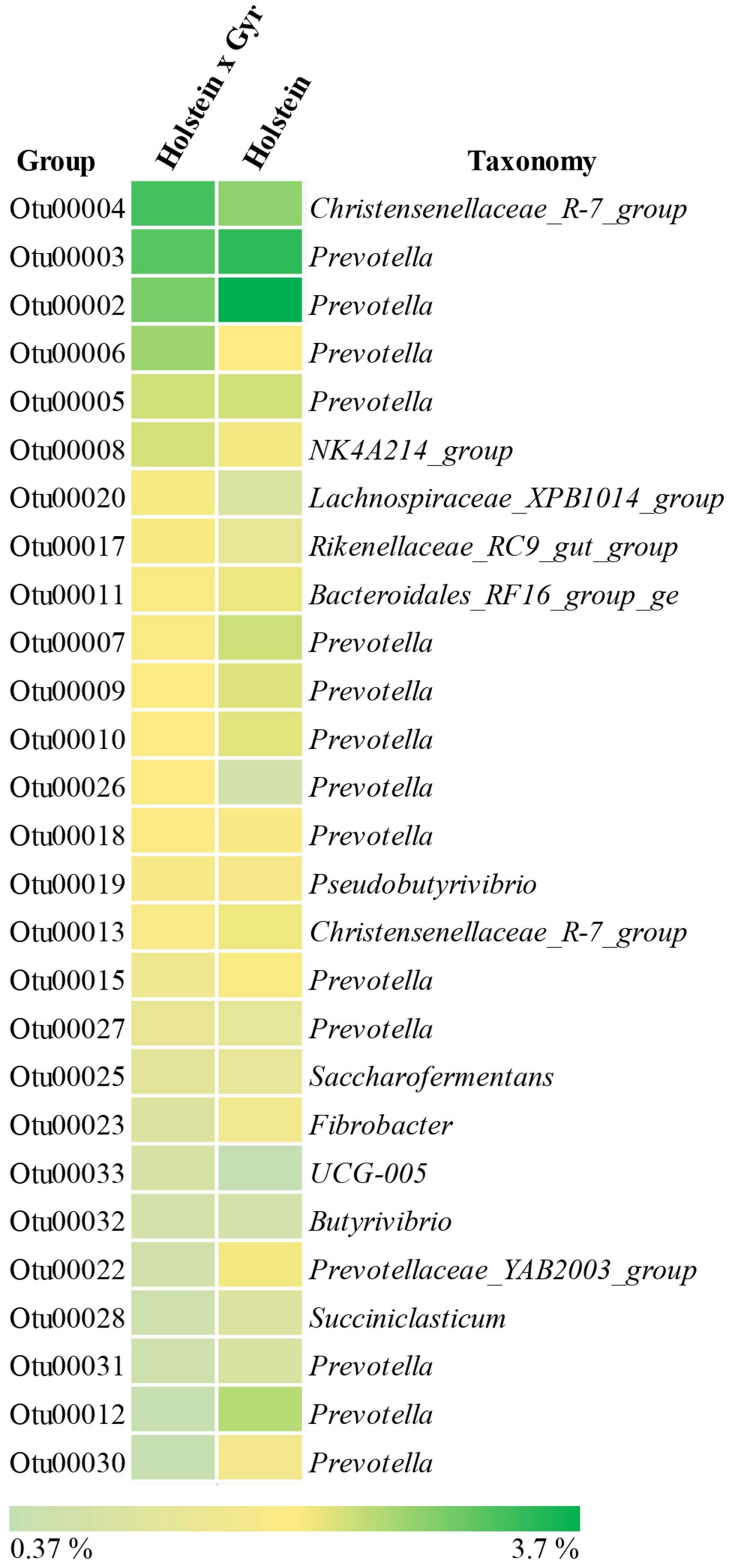
Heatmap of OTUs shared between Holstein × Gyr and Holstein with relative abundance above 0.5%.

The two breeds shared 4,753 OTUs, while 1,782 OTUs were observed only in the Holstein × Gyr breed and 1,640 OTUs were unique to the Holstein breed (Figure 3). Of the 4,753 OTUs shared between the two breeds, the most abundant (>0.5% relative abundance) are represented in Figure 4. OTU 0002 was found in highest abundance (3.7% relative abundance in the Holstein breed, and 2.3% in the Holstein × Gyr breed) and was classified as a member of the genus *Prevotella*. Members of the genus *Prevotella* also represented the most abundant OTUs shared by both breeds (14 OTUs out of 27 OTUs) and differences in relative abundances were detected for this genus between the two animal groups (Figure 4).

The 10 most abundant unique OTUs identified in each breed are listed in Supplementary Table S3, which includes several OTUs of the genus *Prevotella*. The OTUs classified as Prevotellaceae UCG-003 (0.08% ± 0.37%), *Prevotella* (0.07% ± 0.16%), and Bacteroidata unclassified (0.05% ± 0.24%) were found exclusively in Holstein × Gyr heifers (Supplementary Table S3). The unique OTUs identified in Holstein heifers were classified as Prevotellaceae unclassified (0.02% ± 0.06%), Lachnospiraceae unclassified (0.01% ± 0.03%), and *Prevotella* (0.01 ± 0.04%; Supplementary Table S3).

**Figure 5:**
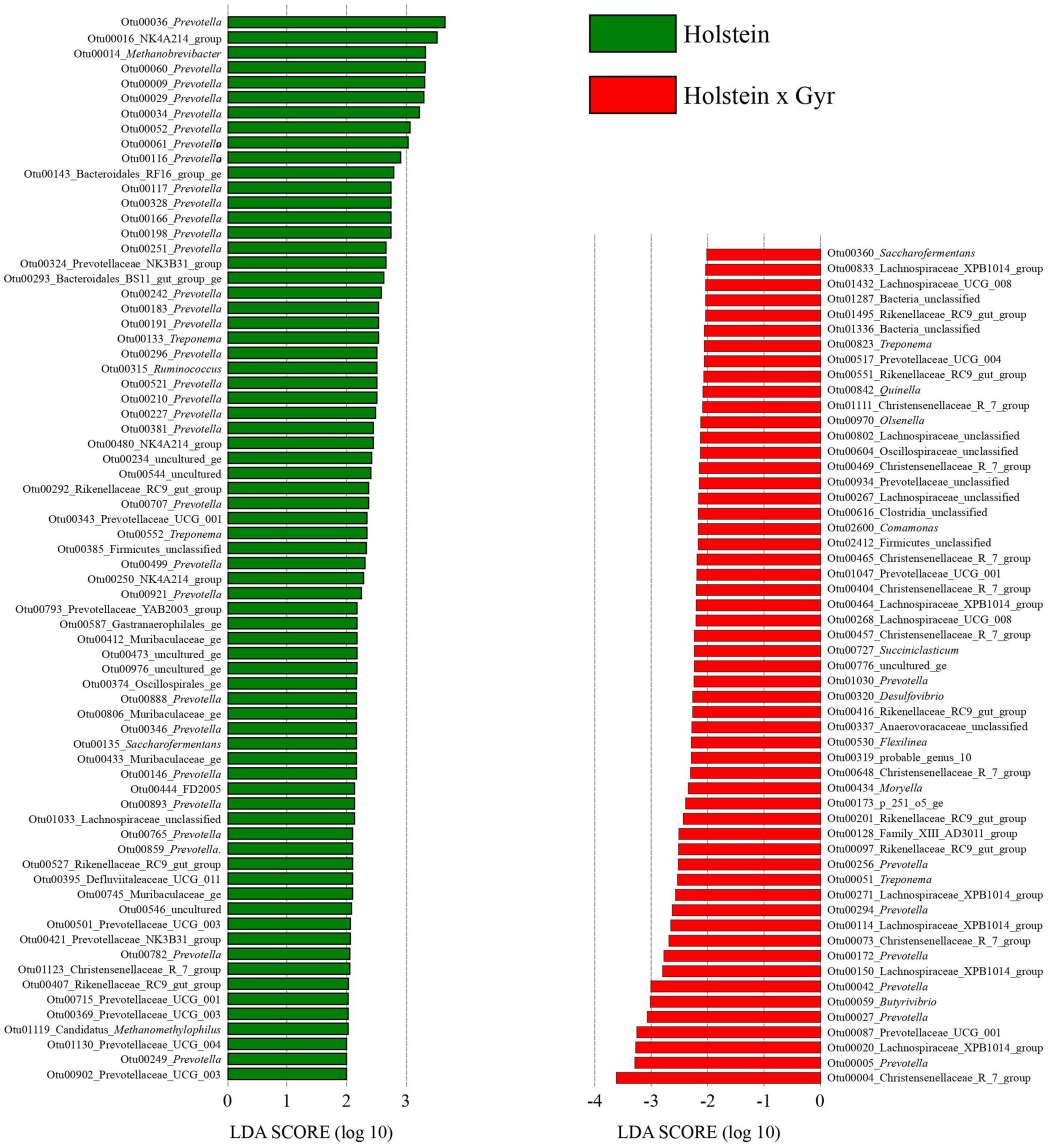
The plot was generated using the online LEfSe project. The length of the bar column represents the LDA score. The figure shows the microbial taxa with significant differences between the Holsteins × Gyr (green) and Holstein (red; LDA score > 2).

The OTUs that could explain the differences associated with Holstein × Gyr steers through LDA analysis, were OTU00004 (Christensenellaceae_R_7_group), OTU00005 (*Prevotella*), and OTU00020 (Lachnospiraceae_XPB1014-group). The OTUs that were considered indicators of the microbiota in Holsteins steers were OTU00036 (*Prevotella*), OTU00016 (NK4A214_group), and OTU00014 (*Methanobrevibacter*; Figure 5).

### 3.4 Tick incidence and polymorphisms in the *IL-1β* gene

**Figure 6:**
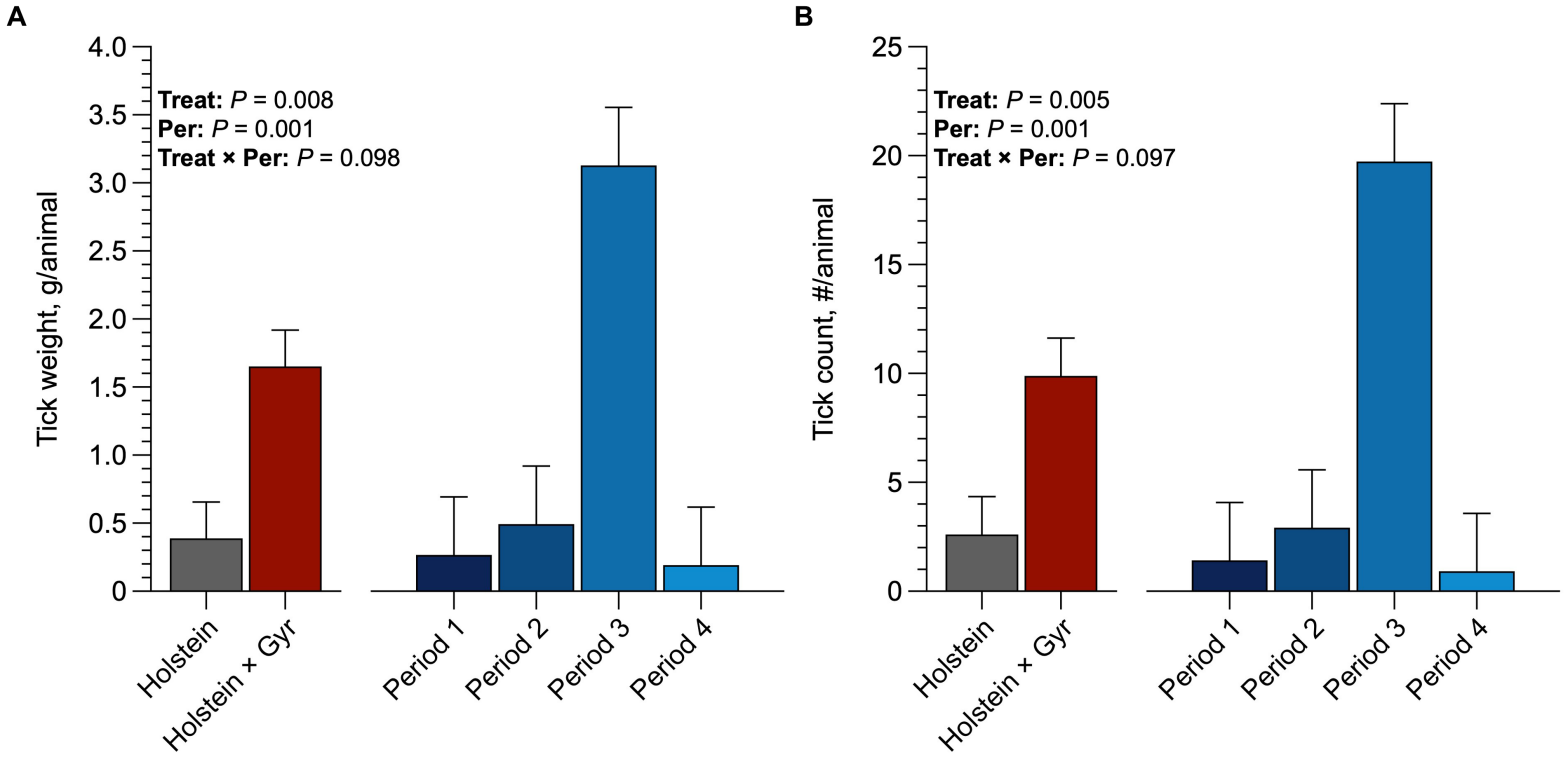
**(A)** Average tick count (number/animals) per breed and average per period of Holstein × Gyr and Holstein heifers in pasture of Guinea grass (*Panicum maximum* Jacq. cv. Mombaça). **(B)** Average tick weight (g/animals) per breed and average per period of Holstein × Gyr and Holstein heifers in pasture of Guinea grass (*Panicum maximum* Jacq. cv. Mombaça).

Holstein heifers had a greater (*p* = 0.06; 9.8 ticks/animal) tick count than the Holstein × Gyr heifers (2.56 ticks/animal; Figure 6). Holstein heifers also had greater tick weight than Holstein × Gyr heifers (1.6 g/animal and 0.4 g/animal; *p* < 0.05). All heifers had more tick infestation in the third period (*p* < 0.01), resulting in greater tick weight (*p* < 0.01).

No mutations were identified at exon 1 of the gene *IL-1β* among 11 sequences analyzed in this study (five Holstein × Gyr and six Holstein heifers). However, mutations were found (Supplementary Figure 5) at Intron I in both breeds. At exon II, we confirmed the mutations described in the reference sequence from GenBank (AY851162.1) for eight of the analyzed sequences (four Holstein × Gyr and four Holstein; Table 2). For Holstein × Gyr heifers the higher presence of nucleotide variation was found in *IL-1β* at base position 2,126 of exon II. Holstein had the most variation at base position 2,184.

**Table 2:** Mutations found in *IL-1β* gene in grazing heifers.

Base position	Holstein × Gyr		Holstein		Reference Sequence (*IL-1β* gene)
Intron 1	Nucleotide	*N*	Nucleotide	*N* ^a^	
1,148	A	3	A	3	G^b^
	G	1	R	3	
1,235	T	3	T	3	C^c^
	Y	2	Y	3	
1,266	G	4	G	3	A^d^
	R	1	R	2	
1,378	C	3	C	3	T^e^
	Y	1	Y^j^	2	
**Exon 2**					
2,126	A	1	-	-	R^f^
	G	2	G	2	
	R	2	-	-	
2,184	G	4	G	4	K^g^
	R	1	R	2	
2,195	G	4	G	4	K
2,209	C	4	C	4	M^h^
2,253	C	4	C	4	S^i^

Despite such nucleotide variants, no differences in the aminoacidic composition were found. ^a^N, number of animals. ^b^A, adenine. ^c^G, guanine. ^d^C, cytosine. ^e^T, tymine. ^f^R, purine nucleotide. ^g^K, nucleotide might be T or G. ^h^M, nucleotide might be C or A. ^i^S, nucleotide might be C or G. ^j^Y, pyrimidine nucleotide.

### 3.5 Blood parameters

The serum urea content (*p* = 0.01), IGF-1 (*p* < 0.01), T3 (*p =* 0.02), T4 (*p* = 0.02), and Albumin (*p* < 0.01) were greater in Holstein × Gyr than in Holstein heifers (Table 3). Breed did not influence serum concentrations of glucose (*p* = 0.35), total protein (*p* = 0.25), or triglycerides (*p* = 0.16).

**Table 3:** Blood parameters of Holstein × Gyr and Holstein heifers under grazing conditions.

Item	Breed			*P*
	Holstein × Gyr	Holstein	SEM^a^	
Urea, mg/dL	29.82	22.70	1.780	0.014
Glucose, mg/dL	71.67	69.78	4.485	0.352
Total protein, g/dL	6.73	7.22	0.183	0.253
Albumin, g/dL	3.29	2.90	0.165	0.001
Triglycerides, mg/dL	30.07	24.25	2.783	0.162
IGF 1, ng/mL	270.67	165.94	39.933	0.001
Total T3, ng/dL	2.73	1.43	0.517	0.016
Total T4, μ/dL	9.98	5.51	1.677	0.016

^a^SEM, standard error of mean.

## 4 Discussion

The present study was conducted to investigate factors contributing to the better performance of Holstein × Gyr heifers compared to Holstein heifers under tropical pastures. Previously, Quirino et al. (2022) highlighted greater average daily gain and feed efficiency for Holstein × Gyr than Holstein heifers managed in an intermittent grazing system of Guinea grass (*Panicum maximum* Jacq. cv. Mombaça). Here, we evaluated differences in rumen microbiota composition, susceptibility to ticks, *IL-1β* nucleotide polymorphisms, and metabolic profile on blood samples aiming to identify factors explaining the better performance of Holstein × Gyr when grazing tropical pastures.

Significant differences in OTU abundance and membership were observed in the rumen microbial community of Holstein × Gyr and Holstein heifers. One OTU classified as *Prevotella* showed greater relative abundance in Holstein heifers. Although most of the OTUs were shared by both breeds, 27.3% of the OTUs detected in Holstein × Gyr heifers were found exclusively in this group. Similarly, 25.1% of the OTUs present in the rumen of Holstein heifers were not found in Holstein × Gyr. Notably, most observed differences were related to the phylum Bacteroidota and OTUs classified as members of the family Prevotellaceae and the genus *Prevotella*. In both Holstein x Gyr and Holstein animals, a large number of exclusive OTUs belonging to the genus *Prevotella* were observed and similar results were obtained in the analysis of indicator OTUs. Although amplicon sequencing has limited resolution to identify taxa at the species level, differences between breeds could be linked to *Prevotella* strains performing distinct functions in the rumen ecosystem of these animals.

*Prevotella* is a bacterial genus generally found in high abundance in the rumen of cattle (Thoetkiattikul et al., 2013; Pandit et al., 2018), probably due to its high genetic and functional diversity, which allows the colonization of various ecological niches in the rumen ecosystem (Jami and Mizrahi, 2012). *Prevotella* is metabolically versatile and capable of utilizing a range of substrates present in the rumen (Stevenson and Weimer, 2009; Jewell et al., 2015). *Prevotella* has been associated with greater concentrate intake due to its proteolytic and amylolytic activity in the rumen (McCann et al., 2014a,b), and some strains can also degrade hemicellulose and pectin (Dehority, 1991; Matsui et al., 1998).

Differences in microbial community composition may occur due to several factors, such as diet, heat stress, and management. For example, heat stress is known to affect animal eating habits, which could induce changes in the composition of the rumen microbiota (Uyeno et al., 2010). However, when animals are fed the same diet and subjected to the same management practices differences in rumen microbiota are less likely, as observed for Holstein × Gyr and Holstein heifers in the current study. It should be pointed out, however, that the animal gut microbiota can remain stable over a short period when animals are kept in a controlled environment, but the microbiome composition can vary in the longer term, which is a limitation of animal trials.

Firmicutes and Bacteroidota were the two most abundant phyla in the rumen of Holstein × Gyr heifers. However, the greater abundance of Firmicutes in Holstein × Gyr heifers may result from a compensation mechanism for decreasing *Prevotella* in the rumen (Jami et al., 2014). Prevotellaceae UCG-003, which was found only in Holstein × Gyr, has been associated with an increase in the production of organic acids in the rumen (Chen et al., 2021). An OTU classified as Bacteroidales unclassified was unique to the Holstein × Gyr heifers, and this taxon has been previously associated with ruminal biohydrogenation (Zhang et al., 2014). Also, the Bacteroidota phylum is related to the production of glycoside hydrolases that degrade substrates a range of substrates in the rumen, including cellulose, pectin, and starch (Hernández et al., 2022).

Tick infestation was low in the current study and may not have directly affected animal performance. This is probably because the animals were treated with anti-parasitic at the beginning of the experiment. Even with the same initial tick control, Holstein heifers had higher tick infestation. An even greater difference would probably be expected without preventive treatment of the animals in each group.

The *IL-1β* gene has been associated with inflammatory response and chronic inflammation (Brannan et al., 2014). The nucleotide sequence of this gene was analyzed since it has been associated with a response to tick infestation (Brannan et al., 2014), In addition, its polymorphism had been previously characterized and was confirmed by findings in the current study. According to the Genbank sequence (AY851162.1), we found nucleotide variations in the Bov-tA SINE region, which is embedded in the intronic region. The Bov-tA SINE is a member of the retrotransposon family Bov-tA and is widely distributed in the ruminant genome (Lenstra et al., 1993). Bov-tA is composed of a tRNA-related region at the 5′ end and a Bov-A unit at the 3′ end (Okada and Hamada, 1997; Shimamura et al., 1999). According to Onami et al. (2007), the Bov-AX family is a candidate for use as a polymorphic DNA marker due to its polymorphic nature and abundance. Additionally, Bov-AX loci may be used for breed discrimination. The presence of mutations in the regulatory region of the gene *IL-1β*, confirms the highly polymorphic nature of the interleukin family (Wu and Wang, 2018) and highlights this region as a potential marker for tick infestation. Those mutations might explain the lower tick infestation in Holstein × Gyr heifers in tropical grazing systems, increasing the pro-inflammatory reaction when compared to Holstein. Moreover, the genomic characterization of immunological gene families may contribute to a better understanding of resistance phenotypes, particularly for mutations in conserved regions such as those described here, and may have relevant roles in general adaptability that might be further investigated. The presence of most mutations in exon 2 of the IL*-1β* gene in Holstein × Gyr associated with less tick infestation and tick weight suggested the greater resistance of these animals.

Despite the presence of nucleotide variants in exon two, based on the reference sequence (GenBank AY851162.1), there were no predicted differences in the amino acid composition of the protein, and phenotypic changes are not expected. Such silent mutations confirm the high variability in the *IL-1β* gene and together with the mutations identified in the Bov-tA family is necessary to understand the effect of these nucleotide substitutions on adaptability and parasite resistance in cattle. Also, an analysis of a larger number of animals might determine the haplotype structure of these breeds and pinpoint the role of each genetic group in the formation of crossbreeds and/or synthetic breeds.

Considering the greater forage dry matter intake and neutral detergent fiber intake for Holstein × Gyr heifers (Quirino et al., 2022), we had a greater concentration of serum urea, which may improve urea recycling. The fermentable carbohydrates are responsible for stimulating the transport of urea to the rumen (Nichols et al., 2022). However, the greater serum urea was not combined with glucose availability in the rumen. Therefore, urea cannot be recycled adequately and might not result in differences in total protein.

Spicer et al. (1990) suggested that serum IGF-1 may be affected by environmental conditions because cows in summer had lower milk IGF-1. Besides, Holstein × Gyr heifers presented greater concentrations of IGF-1, T3, and T4 which are associated with metabolic homeostasis and adaptation to the environment. This might indicate a better adaptation to tropical environments and better performance of Holstein × Gyr heifers. The IGF-1 participates in glucose homeostasis, reducing glucose blood levels and utilization by tissues (Kasprzak, 2021). Also, T4 and T3 were greater in Holstein × Gyr and these hormones are involved with body growth, skeletal development, energy metabolism, and temperature homeostasis (Silva et al., 2021). This could help explain why Holstein × Gyr heifers had a higher tolerance to heat stress (Cavalheira et al., 2021). Collectively, our results show that animals with better metabolism dynamics and adaptation to the environment can perform better, even without greater consumption (Quirino et al., 2022).

## 5 Conclusion

The rumen microbiota of Holstein × Gyr and Holstein heifers showed significant differences in community composition mainly associated with the phylum Bacteroidota and the abundance of members of the family Prevotellaceae and the genus *Prevotella*. The Holstein × Gyr heifers had less tick infestation compared to Holstein, which made these animals perform better. Additionally, the greater concentrations of IGF-1, T3, and T4 suggest that Holstein × Gyr heifers have better energy metabolism and thermoregulation and are more adapted to tropical grazing systems. *IL-1β* polymorphisms are still subject to further studies to disentangle the role of different nucleotide substitutions in tick resistance/susceptibility phenotype.

## Data availability statement

The datasets presented in this study can be found in online repositories. The names of the repository/repositories and accession number(s) can be found at: BioProject, PRJNA956552.

## Ethics statement

The animal study was approved by Animal Care and Handling stated in the Universidade Federal de Viçosa guidelines process number 24/2018. The study was conducted in accordance with the local legislation and institutional requirements.

## Author contributions

PR and MM designed the study. DQ, JS, HM, and LR conceived the study. DQ, KO, SG, CC, and PR wrote and edited the manuscript. GS and HM critically reviewed, edited, and finalized the manuscript for submission. All authors contributed to the article and approved the submitted version.

## Footnotes

^1^ http://www.ncbi.nlm.nih.gov/sra

^2^ http://drive5.com/muscle/downloads.htm

## Supplementary material

Supplementary Figure S1 Non-metric Multidimensional Scaling (NMDS) using Bray-Curtis dissimilarity as distance metric for OTUs in ruminal samples of Holstein × Gyr and Holstein heifers. The data in the upper panel **(A)** is representative of the three sampling periods combined while the lower panels **(B)** represent separate sampling periods.

Supplementary Figure S2 Alpha diversity indices of the ruminal samples of Holstein × Gyr and Holstein heifers (mean and standard deviation).

Supplementary Figure S3 Bacterial community composition at the family level (relative abundances > 1.0%) in ruminal samples of Holstein × Gyr and Holstein heifers.

Supplementary Figure S4 Bacterial community composition at the genus level (relative abundances > 1.0%) in ruminal samples of Holstein × Gyr and Holstein heifers.

Supplementary Figure S5 *IL1 β* intron 1 sequencing results. **(A)** The arrow indicates mutation in base number 1,148. **(B)** The arrow indicates mutation in base number 1,235. **(C)** The arrow indicates mutation in base number 1,266. **(D)** The arrow indicates mutation in base number 1,378.
