# Supplementary material for: Comparison of ruminal microbiota, IL-1β gene variation, and tick incidence between Holstein × Gyr and Holstein heifers in grazing system
Source: Front Microbiol. 2024 Feb 26;15:1132151. doi: 10.3389/fmicb.2024.1132151 (PMC10925795; doi:10.3389/fmicb.2024.1132151)
Supplement: Supplementary file 1 [file Table_1.DOCX]

**S. TABLE 1** Ingredients and composition of supplement offered to heifers.

| Ingredients | % Dry matter |
| --- | --- |
| Soybean meal | 22.0 |
| Cornmeal | 75.0 |
| Urea | 2.7 |
| Ammonium sulfate | 0.3 |
