# Supplementary material for: Comparison of ruminal microbiota, IL-1β gene variation, and tick incidence between Holstein × Gyr and Holstein heifers in grazing system
Source: Front Microbiol. 2024 Feb 26;15:1132151. doi: 10.3389/fmicb.2024.1132151 (PMC10925795; doi:10.3389/fmicb.2024.1132151)
Supplement: Supplementary file 2 [file Table_2.DOCX]

**S. TABLE 2** Nucleotide sequences of primers used in the PCR reactions.

| Gene symbol | Primer sequence (5’-3’) | Aces. Number NCBI | |
| --- | --- | --- | --- |
| *IL-1β* |  | | AY851162.1 |
| Exon 1 | Fw^a^: CTGCAGTGCCATATCCCTGA | |  |
|  | Rev^b^: GTGGTTTTGGGAGTGCAGTG | |  |
| Exon 2 | Fw: TTTGCTGCAGGAAGTAGACCC | |  |
|  | Rev: CAGATCCAAGAAAAGTCAATGACCA | |  |

^a^ Fw, forward. ^b^ Rev, reverse.
