# Supplementary material for: Comparison of ruminal microbiota, IL-1β gene variation, and tick incidence between Holstein × Gyr and Holstein heifers in grazing system
Source: Front Microbiol. 2024 Feb 26;15:1132151. doi: 10.3389/fmicb.2024.1132151 (PMC10925795; doi:10.3389/fmicb.2024.1132151)
Supplement: Supplementary file 3 [file Table_3.docx]

**S. TABLE 3** List of the 10 most abundant unique OTUs present in ruminal samples of Holstein × Gyr and Holstein heifers on 3 sampling days.

| Holstein × Gyr | | | | | | |
| --- | --- | --- | --- | --- | --- | --- |
| OTU | Relative abundance (%) | Standard deviation (%) | *P*-values | | *P*-value (FDR) | Taxonomy |
| Otu00652 | 0.0784 | 0.3657 | 0.0010 | | 0.0446 | Prevotellaceae_UCG-003 |
| Otu00556 | 0.0711 | 0.1593 | 0.0010 | | 0.0439 | *Prevotella* |
| Otu00497 | 0.0541 | 0.2405 | 0.0010 | | 0.0421 | Bacteroidales_unclassified |
| Otu00520 | 0.0535 | 0.2566 | 0.0010 | | 0.0614 | *Prevotella* |
| Otu00895 | 0.0384 | 0.1156 | 0.0010 | | 0.0383 | Prevotellaceae_UCG-003 |
| Otu01015 | 0.0312 | 0.0509 | 0.0010 | | 0.0478 | Bacteroidales_uncultured |
| Otu01458 | 0.0260 | 0.1086 | 0.0060 | | 0.1172 | *Prevotella* |
| Otu01267 | 0.0211 | 0.0781 | 0.0010 | | 0.0504 | Prevotellaceae_UCG-003 |
| Otu01408 | 0.0206 | 0.0589 | 0.0010 | | 0.0451 | Prevotellaceae_UCG-003 |
| Otu01534 | 0.0182 | 0.0411 | 0.0020 | | 0.0563 | *Prevotella* |
| Holstein | | | | | | |
| OTU | Relative abundance (%) | Standard deviation(%) | *P*-value | *P*-value (FDR) | | Taxonomy |
| Otu01040 | 0.0215 | 0.0582 | 0.0090 | 0.1408 | | Prevotellaceae_unclassified |
| Otu01479 | 0.0141 | 0.0323 | 0.0020 | 0.0638 | | Lachnospiraceae_unclassified |
| Otu01709 | 0.0123 | 0.0397 | 0.0010 | 0.0406 | | *Prevotella* |
| Otu01832 | 0.0074 | 0.0234 | 0.0010 | 0.0370 | | *Muribaculaceae*_ge |
| Otu02270 | 0.0066 | 0.0318 | 0.0010 | 0.0556 | | Bacteria_unclassified |
| Otu02348 | 0.0066 | 0.0148 | 0.0050 | 0.0998 | | Lachnospiraceae_unclassified |
| Otu02606 | 0.0058 | 0.0224 | 0.0010 | 0.0419 | | *Prevotella* |
| Otu02341 | 0.0057 | 0.0186 | 0.0060 | 0.1145 | | *Anaeroplasma* |
| Otu02881 | 0.0057 | 0.0262 | 0.0010 | 0.0449 | | *Prevotella* |
| Otu03075 | 0.0049 | 0.0189 | 0.0010 | 0.0417 | | *Prevotella* |
|  |  |  |  |  | |  |
