# Supplementary figures and images for: Comparison of ruminal microbiota, IL-1β gene variation, and tick incidence between Holstein × Gyr and Holstein heifers in grazing system
Source: Front Microbiol. 2024 Feb 26;15:1132151. doi: 10.3389/fmicb.2024.1132151 (PMC10925795; doi:10.3389/fmicb.2024.1132151)

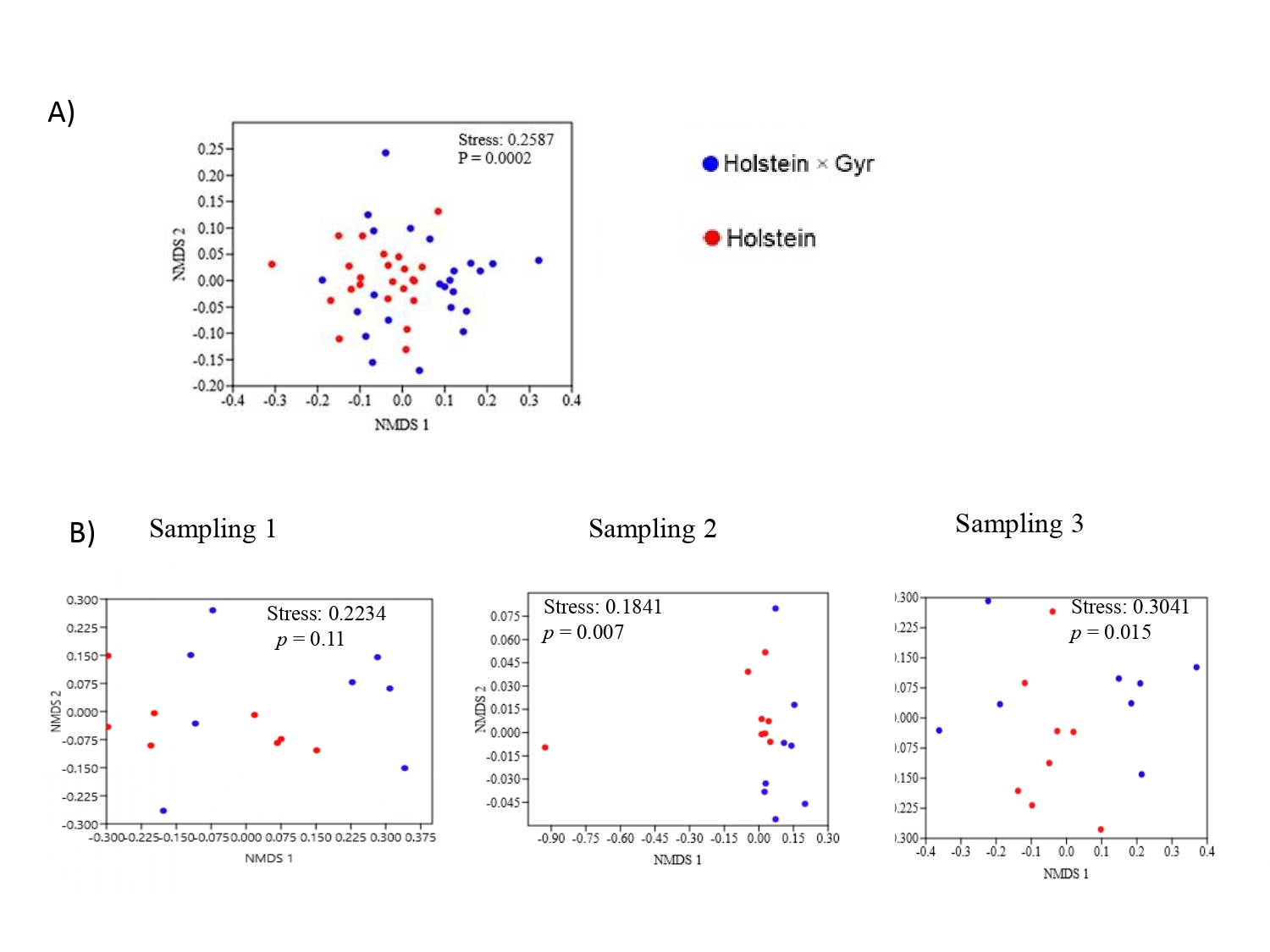

Supplement: Supplementary Figure S1 — Non-metric Multidimensional Scaling (NMDS) using Bray-Curtis dissimilarity as distance metric for OTUs in ruminal samples of Holstein × Gyr and Holstein heifers. The data in the upper panel (A) is representative of the three sampling periods combined while the lower panels (B) represent separate sampling periods. [file Image_1.JPEG]

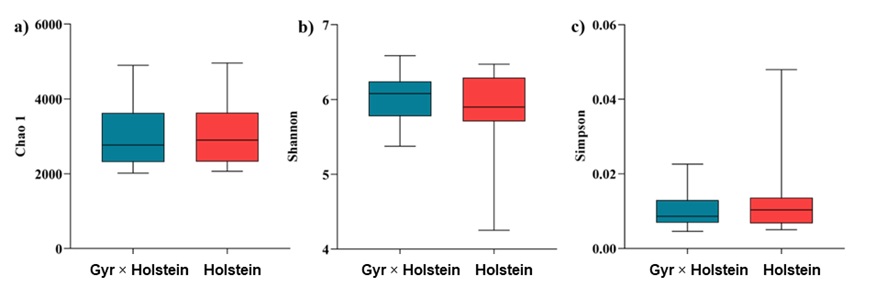

Supplement: Supplementary Figure S2 — Alpha diversity indices of the ruminal samples of Holstein × Gyr and Holstein heifers (mean and standard deviation). [file Image_2.JPEG]

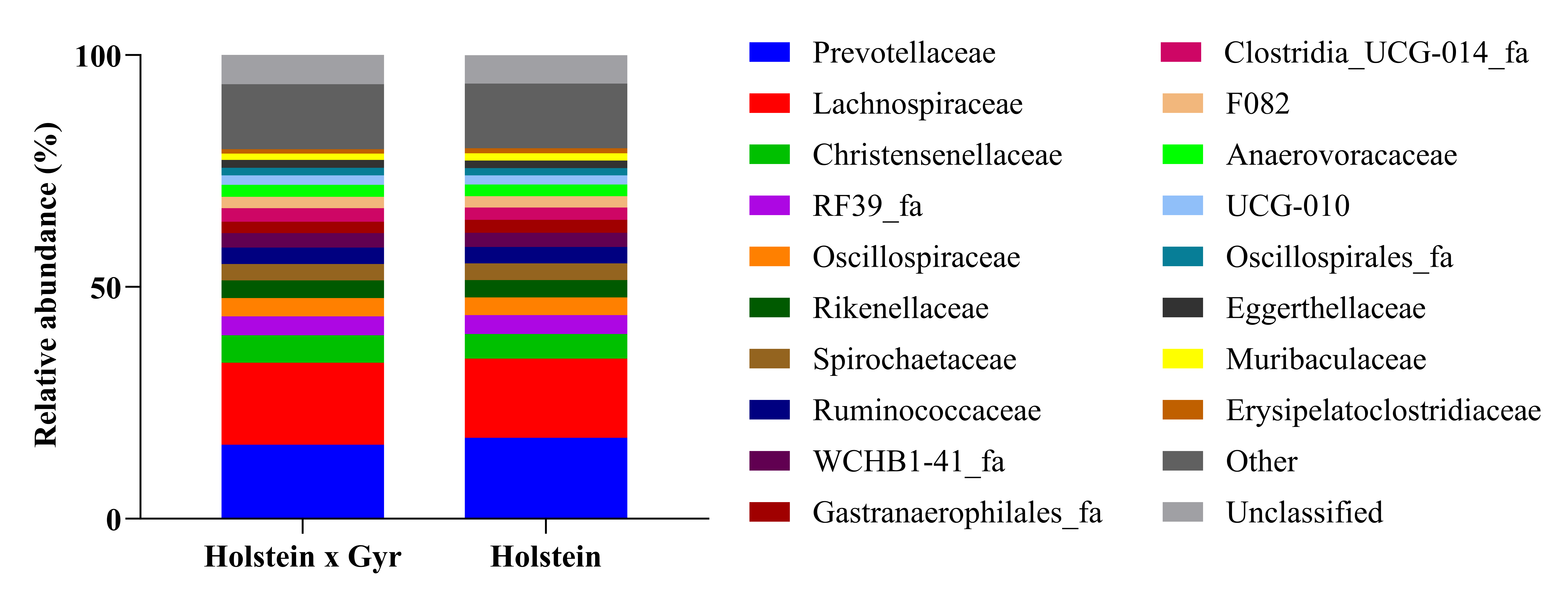

Supplement: Supplementary Figure S3 — Bacterial community composition at the family level (relative abundances > 1.0%) in ruminal samples of Holstein × Gyr and Holstein heifers. [file Image_3.TIF]

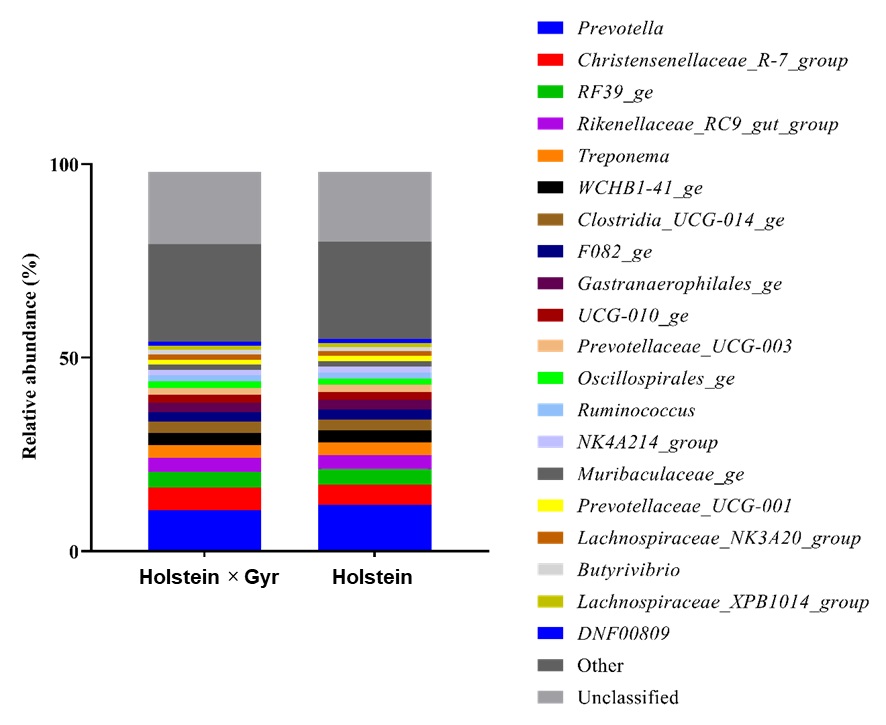

Supplement: Supplementary Figure S4 — Bacterial community composition at the genus level (relative abundances > 1.0%) in ruminal samples of Holstein × Gyr and Holstein heifers. [file Image_4.JPEG]

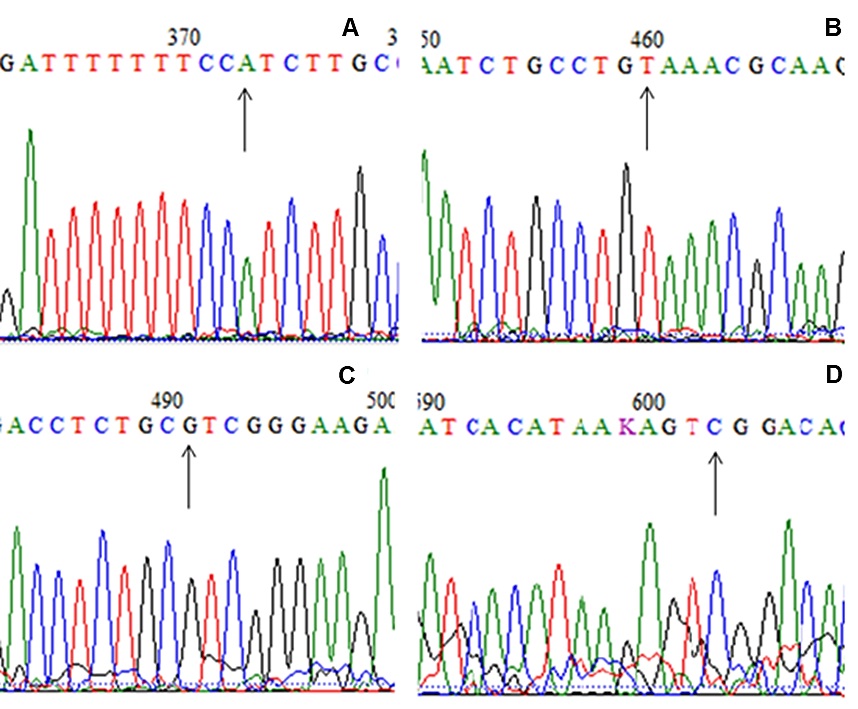

Supplement: Supplementary Figure S5 — IL1 β intron 1 sequencing results. (A) The arrow indicates mutation in base number 1,148. (B) The arrow indicates mutation in base number 1,235. (C) The arrow indicates mutation in base number 1,266. (D) The arrow indicates mutation in base number 1,378. [file Image_5.JPEG]
